# Angiotensin II Type 1 Receptor (AT-1R) Expression Correlates with VEGF-A and VEGF-D Expression in Invasive Ductal Breast Cancer

**DOI:** 10.1007/s12253-012-9516-x

**Published:** 2012-05-12

**Authors:** Aleksandra Jethon, Bartosz Pula, Aleksandra Piotrowska, Andrzej Wojnar, Janusz Rys, Piotr Dziegiel, Marzena Podhorska-Okolow

**Affiliations:** 1 Department of Histology and Embryology, Medical University, Chalubinskiego 6a, 50-368, Wroclaw, Poland; 2 Department of Pathomorphology, Lower Silesian Centre of Oncology, Wroclaw, Poland; 3 Department of Tumour Pathology, M. Sklodowska-Curie Institute, Centre of Oncology, Cracow, Poland; 4 Department of Histology and Embryology, Poznan University of Medical Sciences, Poznan, Poland

**Keywords:** Angiotensin receptors, VEGF, Angiogenesis, Lymphangiogenesis, Breast cancer

## Abstract

Recent studies point to the involvement of angiotensin II (Ang II) receptor type 1 (AT-1R) on processes of metastasing, stimulation of invasiveness and angiogenesis in tumours. In this study, the correlation between intensity of AT-1R expression and expression of lymph- and angiogenesis markers in invasive ductal breast cancers (IDC) was examined. Immunohistochemical studies (IHC) were performed on archival material of 102 IDC cases. Only 28 (27.5%) cases manifested low AT-1R expression while 74 (72.5%) cases demonstrated a moderate or pronounced AT-1R expression. Expression intensity of AT-1R was found to correlate with expressions of VEGF-A (*r* = 0.26; *p* = 0.008) and VEGF-D (*r* = 0.24; *p* = 0.015). Out of the examined markers of angiogenesis and lymphangiogenesis only the pronounced expression of VEGF-C was found to correlate with patient poor clinical outcome (*p* = 0.009). The positive correlation between AT-1R and VEGF-A and VEGF-D could point to stimulatory action of Ang II on their expression what might result in augmented lymph- and angiogenesis in IDC.

## Introduction

Breast cancer is the most frequent female malignancy all over the world. In 2008 its incidence was appraised at 1.38 million new cases (23% of all tumours) with increasing frequency of its manifestation [1, 2].

Angiotensin II (Ang II) represents an important element of renin-angiotensin system (RAS). It is a multifunctional octapeptide, formed by excision of two amino acids (histidine and leucine) from carbonyl terminus of its precursor, angiotensin I. The reaction is executed by angiotensin converting enzyme (ACE), secreted mainly by the endothelium of pulmonary blood vessels [3]. Ang II acts *via* two types of receptors, AT-1R and AT-2R, which are coupled to G proteins [4]. Ang II stimulates the release of aldosteron, vasopressin and nitrogen oxide. It is involved in the development of oxidative stress, inflammatory processes, maintenance of an appropriate level of body electrolytes and water, in blood vessel remodelling, processes of vascular atherogenesis and it stimulates the contraction of vascular myocytes [5–7]. Expression of AT-1R was demonstrated in cells of several organs [8]. On the other hand, angiotensin type 2 receptor (AT-2R), responsible, *i.a.*, for induction of apoptosis, is less common and expressed mainly in fetal cells. In an adult healthy organism, it is expressed in cells of vascular endothelium, uterine muscle, ovaries, some cerebral structures and in selected structures of skin, kidneys, heart [9, 10].

Several earlier studies pointed to a significant function of Ang II on several aspects of neoplastic disease and compounds suppressing action of Ang II were suggested to have an anti-neoplastic significance [11]. Overexpression of AT-1R was observed in many types of tumours including tumours of pancreas, kidneys and lungs [12–14].

In mammary gland, presence of both receptor types was demonstrated [8]. AT-1R is located in epithelial cells of alveoli and lactiferous ducts, both in the healthy tissue and in benign tumours (15). Increased levels of AT-1R mRNA were observed in hyperplastic lesions, in ductal breast cancer in situ (DCIS) and in IDC [16]. AT-1R activation by Ang II resulted in proliferation of breast cancer cells [7, 17]. In cases of metastazing IDC only a weak cytoplasmic expression of AT-1R was demonstrated [6]. The till now performed studies showed also that breast cancer cells may acquire metastazing ability *i.a*. due to changes in activity and accessibility of Ang II in the pathway linked to AT-1R [18, 19]. It was also shown that blockade of AT-1R decreased invasiveness of tumour cells [20]. No such effect was shown in the case of AT-2R. The results of the studies mentioned above point to a potential of applying AT-1R blockers in anti-neoplastic therapy [19].

Ang II is also engaged in the process of angiogenesis, of key importance from the point of view of neoplastic progression [21, 22]. In numerous studies stimulatory effect of Ang II on the formation of new blood vessels was shown [23]. Moreover, blockade of ACE and AT-1R significantly inhibited development of many tumour types (breast cancer, non-small cell pulmonary cancer, gastric cancer) and decreased the density of blood vessels as well as their metastatic potential [12, 24]. In the case of tumour lymphangiogenesis no data are currently available on the involvement of Ang II.

In this study, we undertook an attempt to define the significance of AT-1R expression, with particular attention paid to its correlation with selected markers of angiogenesis (CD31, VEGF-A) and lymphangiogenesis (D2-40, Lyve-1, VEGF-C and VEGF-D) in IDC.

## Material and Methods

### Patients

The studies were conducted on archival paraffin blocks of IDC subjected to radical surgery in 1999–2002 in the Lower Silesia Centre of Oncology in Wroclaw. Clinical and pathological data were obtained from hospital archives. A group of 102 patients, aged between 30 and 83 years (mean of 56 years) was retrospectively analyzed. Duration of observation ranged between 1 and 125 months (mean of 61 months). The clinical and pathological data are summarized in Table 1.

**Table 1:** Clinical and pathological data of 102 studied cases of IDC

Parameter	Number of cases	%
Patient’s age		
≤ 50	35	34.3
> 50	67	65.7
Menopause		
Pre	38	37.3
Post	64	62.7
Tumour size		
≤ 2 cm	64	62.7
> 2 cm	38	37.3
Grade of malignancy		
G1	10	9.8
G2	57	55.9
G3	35	34.3
pT		
1	59	57.8
2	32	31.5
3	8	7.8
4	3	2.9
pN		
pN0	52	51
pN1-3	50	49
ER		
Positive	81	79.4
Negative	21	20.6
PR		
Positive	68	66.7
Negative	34	33.3
HER2 (IHC)		
Positive	15	14.7
Negative	87	85.3
Ki-67		
≤ 25%	76	74.5
> 25%	26	25.5

### Immunohistochemistry (IHC)

Immunohistochemical reactions were performed on paraffin sections using DAKO Autostainer Link48 (Dako, Glostrup, Denmark) in case of the following antibodies in respective concentrations: mouse anti-AT-1R (24.7 μg/ml; Abcam, Cambridge, UK), mouse anti-CD31 (0.69 μg/ml; Leica Microsystems, Wetzlar, Germany), mouse anti-D2-40 (RTU; Dako), mouse anti-VEGF-A (1 μg/ml; Dako) and mouse anti-Ki-67 (clone MIB-1, 0.8 μg/ml; Dako). In order to deparaffinize, rehydrate and unmask the antigens the sections were boiled in Target Retrieval Solution buffer (pH 9; Dako) using Pre-Treament Link Platform (Dako) and, subsequently, cooled in a rinsing buffer (TBS). EnVision™ FLEX (Dako) was used to visualize the antigens. Only in the case of VEGF-A IHC reaction, incubation with primary antibodies was extended from 20 min at RT to 18 hours at the temperature of 4°C and reaction was amplified using the EnVision™ FLEX + Mouse LINKER system (Dako).

Reactions with ER-specific antibodies (detecting estrogen receptors), PR-specific antibodies (detecting progesteron receptors) and with HER-2-specific antibodies were conducted according to the earlier described procedure [25]. The sections were deparaffinized and rehydrated in Antigen Retrieval Solution (pH 6; Dako). Activity of endogenous peroxidase was blocked by 5 min incubation in 3% H_2_O_2_. Subsequently, primary antibodies to ER (clone 1D5, 1:100) or PR (clone PgR 636, 1:100) and incubated for 1 h at RT. The subsequent reaction stages followed manufacturer’s instructions for the LSAB + System-HRP visualization system (Dako). 3,3′-diaminobenzidine (DAB) was used as a chromogen. In order to visualize expression of HER2, the HercepTest™ kit (Dako) was used, according to the procedure recommended by the producer.

In order to examine the expression of VEGF-C, VEGF-D and Lyve-1, mouse antibodies anti-VEGF-C (2 μg/ml), anti-VEGF-D (2 μg/ml) and anti-Lyve-1 (1 μg/ml) were used (ReliaTech GmbH, Braunschweig, Germany). The sections were deparaffinized in xylene, rehydrated and boiled in a citrate buffer, pH 6 (for VEGF-C, Lyve-1) or pH 9 (for VEGF-D). Subsequently, activity of endogenous peroxidase was blocked by 5 min incubation in 3% H_2_O_2_. The sections were incubated with primary antibody overnight at the temperature of 4°C (VEGF-C, VEGF-D) or for 30 min at room temperature (Lyve-1). Then the antigens were visualized using the EnVision™ Detection Systems Peroxidase/DAB, Rabbit/Mouse (Dako).

All slides were counterstained with Mayer’s hematoxylin (Dako). Subsequently, the preparations were mounted in SUB-X Mounting Medium (Dako).

### Evaluation of IHC Reactions

In all cases hematoxylin-eosin (H + E) staining of the preparations was performed in order to determine grade of tumour (G) and to evaluate the extent of necrosis in the tumours.

The intensity of IHC reactions for AT-1R, VEGF-A, VEGF-C and VEGF-D was estimated using the semi-quantitative immunoreactive (IRS) scale of Remmele and Stegner [26]. Determination of Ki-67 antigen expression employed a five grade scale, reflecting percentage of tumour cells manifesting a nuclear reaction: 0% - 0, 1–10% - 1, 11–25% - 2, 26–50% - 3, 51–100% - 4. For quantification of blood vessel and lymphatic vessel densities in the tumour and in the peritumoural area (CD31^+^, Lyve-1^+^ and D2-40^+^) Chalkley point array (Pyser Sgi., Edenbridge, UK) was used. Under magnification of 200x, the vessels were scored in three intratumoural and peritumoural areas of potentially the highest density (hot spots). All the preparations were examined using OLYMPUS BX-41 light microscope (Olympus, Tokyo, Japan).

### Statistical Analysis

The results were subjected to statistical analysis using Prism 5.0 (GraphPad, CA, USA). Relationships between expression of studied markers and clinicopathological factors were examined using the Spearman rank correlation test, the Kruskal-Wallis test and the exact Fisher’s test. Kaplan-Meier’s survival curves for the patients survival were analysed using the test of Mantel Cox. The differences were regarded significant at *p* < 0.05.

## Results

### Expression of AT-1R in Ductal Breast Cancer and its Correlation with Clinical and Pathological Data

**Fig. 1:**
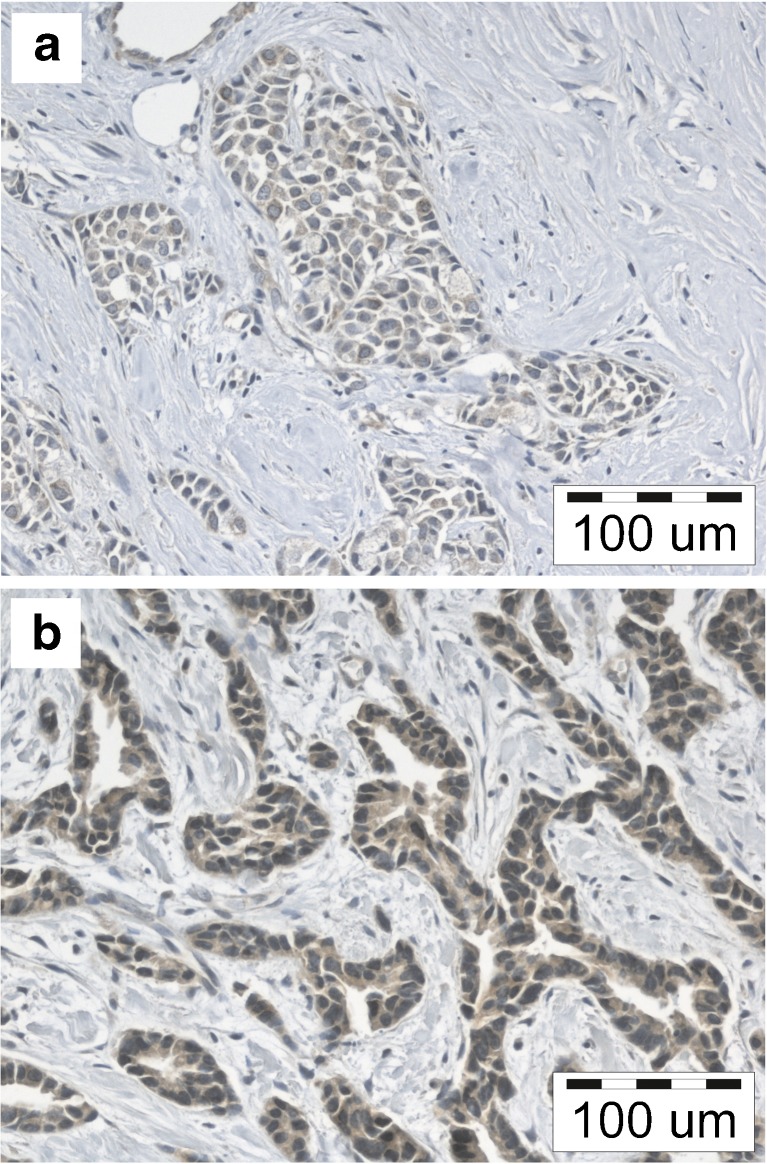
IDC cases with low (**a**) or high (**b**) expression of AT-1R. Magnification 200x

In most cases (101; 99.1%) cytoplasmic expression of AT-1R was noted. Among them, 28 (27.5%) cases demonstrated low expression of AT-1R (IRS 0–4) (Fig. 1a), while 74 (72.5%) cases manifested moderate or high expression of AT-1R (IRS 6–12) (Fig 1b). Expression of AT-1R was detected also in the cells of tumour stroma. No significant differences were disclosed in AT-1R expression, which would be related to degree of the tumour malignancy. Expression of AT-1R failed to correlate with patients’ age, tumour size or presence of metastases to lymph nodes, with expressions of ER, PR or expression of HER2 receptor (Table 2). Expression of AT-1R did not correlate with expression of Ki-67 proliferation antigen (Sperman’s rank correlation test, *p* = 0.85).

**Table 2:** Correlation between AT-1R expression and selected clinical and pathological parameters

Variable	Number of cases (%)	AT-1R—number (%)		*p*
		IRS 0-4	IRS 6-12	
Age				
≤50	35 (34.3)	14 (40.0)	21 (60.0)	0.0601
>50	67 (65.7)	14 (20.9)	53 (79.1)	
Menopause				
Pre	38 (31.3)	13 (34.2)	25 (63.8)	0.2588
Post	64 (68.7)	15 (23.5)	49 (76.5)	
pT				
≤ 2 cm	64 (62.7)	19 (29.7)	45 (70.3)	0.6473
> 2 cm	38 (37.3)	9 (23.6)	29 (76.4)	
pN				
pN0	52 (51.0)	14 (26.9)	38 (73.1)	1.0000
pN1-3	50 (49.0)	14 (28.0)	36 (72.0)	
ER				
Positive	81 (79.4)	22 (27.1)	59 (72.9)	1.0000
Negative	21 (20.6)	6 (28.6)	15 (71.4)	
PR				
Positive	68 (66.7)	20 (29.4)	48 (70.6)	0.6404
Negative	34 (33.3)	8 (23.5)	26 (76.5)	
HER2				
Positive	15 (14.7)	3 (20.0)	12 (80.0)	0.7547
Negative	87 (85.3)	62 (71.2)	15 (13.8)	

### Correlation Between AT-1R Expression and Markers of Angiogenesis and Lymphangiogenesis

All the studied vascular endothelial growth factors (VEGF-A, VEGF-C and VEGF-D) manifested a cytoplasmic reaction in tumour cells. A pronounced expression of VEGF-A was noted in 52 (51%), of VEGF-C in 56 (54.9%), and of VEGF-D in 48 (47.1%) studied cases. A moderately positive correlation was documented between expression of AT-1R on one hand and expression of VEGF-A (*r* = 0.26, *p* = 0.008) and VEGF-D expression (*r* = 0.24, *p* = 0.015) on the other (Table 3).

**Table 3:** Correlation between AT-1R expression and expression of vascular endothelial growth factors (VEGF). Bold numbers indicate a significant correlation

VEGF	r	*p*
VEGF-A	**0.26**	**0.008**
VEGF-C	0.09	0.356
VEGF-D	**0.24**	**0.015**

No correlation was disclosed between the density of CD31^+^ blood vessels expressed intra- and peritumorally and the intensity of AT-1R expression. Also no relationship could be disclosed between the intensity of AT-1R expression and the density of Lyve-1^+^ and D2-40^+^ lymphatic vessels (Table 4).

**Table 4:** Correlation between AT-1R expression and selected markers of vascular density (CD31, Lyve-1, D2-40)

Marker	Number of cases (%)	AT-1R—Number (%)		*p*
		IRS 0-4	IRS 6-12	
CD31 Intra				
≤ 4	24 (23.5)	8 (33.3)	16 (66.7)	0.4475
> 4	78 (76.5)	20 (25.6)	58 (74.4)	
CD31 Peri				
≤ 6	44 (43.1)	14 (31.8)	30 (68.2)	0.5021
> 6	58 (56.9)	14 (24.1)	44 (75.9)	
Lyve-1 Intra				
≤ 1	96 (94.1)	27 (28.1)	69 (71.9)	0.3773
> 1	6 (5.9)	1 (16.7)	5 (83.3)	
Lyve-1 Peri				
≤ 4	77 (75.5)	19 (24.7)	58 (75.3)	0.3068
> 4	25 (24.5)	19 (76.0)	6 (14.0)	
D2-40 Intra				
≤ 2	51 (50.0)	11 (21.6)	40 (78.4)	0.2676
> 2	51 (50.0)	17 (33.3)	34 (66.7)	
D2-40 Peri				
≤ 4	52 (51.0)	12 (23.1)	40 (76.9)	0.3733
> 4	50 (49.0)	16 (32.0)	34 (68.0)	

Intra—intratumoural vessels, Peri—peritumoural vessels

### Effect of AT-1R Expression on Patients’ Outcome

**Fig. 2:**
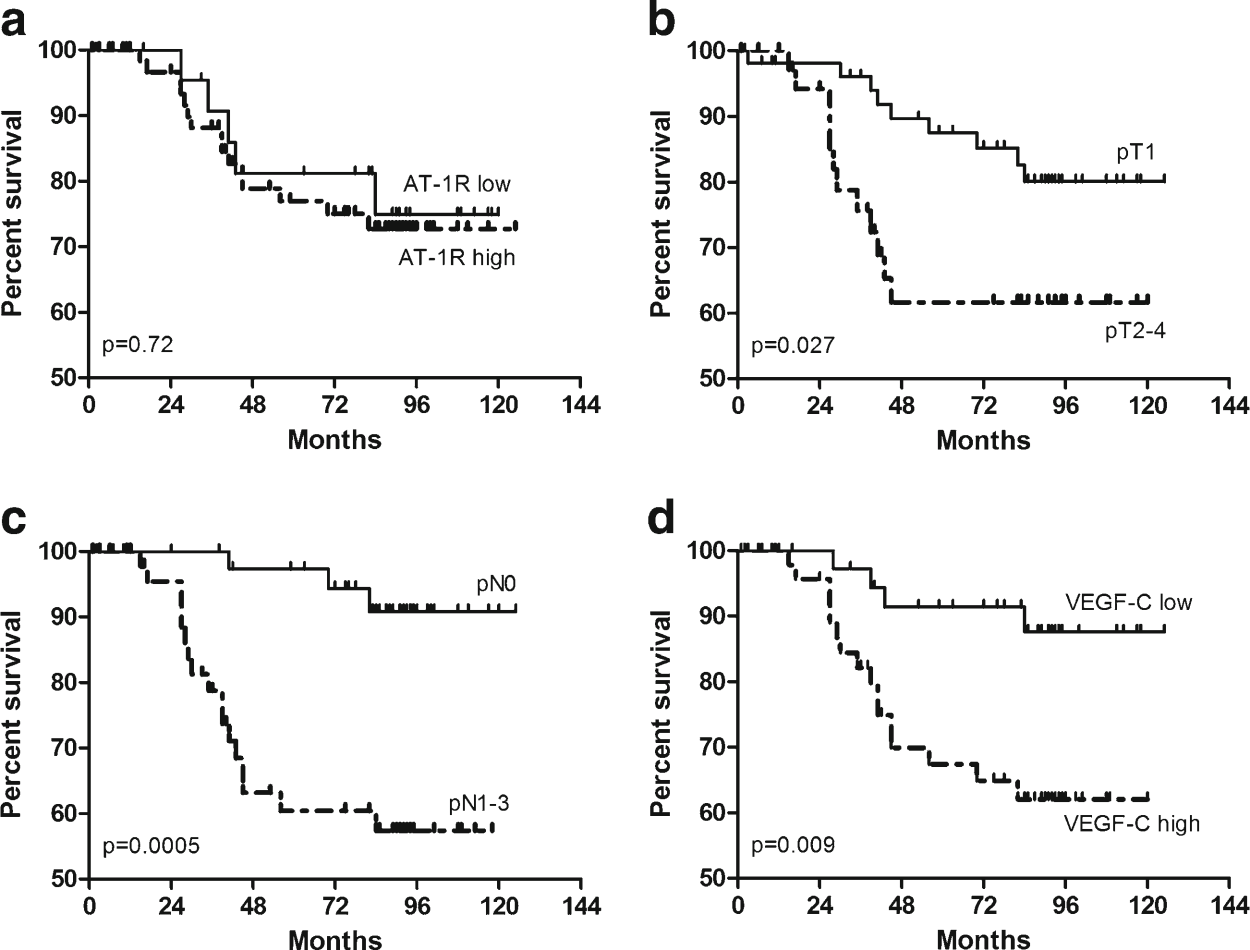
Kaplan-Meier’s diagram of survival for 102 patients, as related to intensity of AT-1R expression (**a**), primary tumour size (**b**), presence lymph node metastases (**c**) and intensity of VEGF-C expression (**d**)

No significant differences were noted in patients survival regarding the intensity of AT-1R expression (Fig. 2a). The variables which significantly affected duration of patients’ survival were primary tumour size (pT) (*p* = 0.028), presence of lymph nodes metastases (pN) (*p* = 0.0005) and high intensity of VEGF-C expression (*p* = 0.009) (Figs. 2b–d, respectively).

## Discussion

Results of earlier studies showed that the function of RAS system is not restricted exclusively to processes controlling body ions turnover but it may play a significant role in processes of proliferation, apoptosis and cell differentiation and significantly affect angiogenesis in neoplastic tumours [27]. Apart from function of the systemic RAS system, activities of a local RAS system have been demonstrated in several types of normal and neoplastic tissues, including tissues and cancers of the breast [8, 15]. Moreover, in malignant lesions an increased AT-1R mRNA expression was demonstrated, as compared to healthy cells of mammary gland [4]. However, a decreased expression of AT-1R protein was detected by some authors in mammary cancers [28]. In turn, studies of Puddefoot et al. demonstrated increased metastatic potential of breast cancer cells dependent on integrin expression changes due to Ang II stimulation [18]. Ang II was also shown to promote cell proliferation by binding to AT-1R, by the activation of the phosphokinase C (PKC), what resulted in calcium ions mobilization [7, 28]. In addition, a stimulatory effect of Ang II on expression of VEGF-A was demonstrated exclusively in triple negative breast cancer (with no expression of ER, PR, HER2), but not on other breast cancer cell lines [19].

Results of our IHC investigations have documented a positive correlation of AT-1R expression intensity with the intensity of VEGF-A expression, corroborating earlier results obtained on other tumours with use of AT-1R blockers, which inhibited growth of tumour cells [20, 27, 29–33]. We have demonstrated also a positive correlation between AT-1R expression and intensity of VEGF-D expression. Earlier results related to the role of VEGF-D expression in neoplastic progression frequently yielded equivocal results [34–36]. According to Gu et al., increased expression of VEGF-D in breast cancer cells correlated with the development of metastases to lymph nodes and patients shorter survival [34]. On the other hand, studies of Gisterek et al., Mohammad et al. and Mylon et al. did not demonstrate any unfavourable effect of VEGF-D expression on patients’ survival [35, 37, 38]. Moreover, the results of studies investigating the relationship between expression of VEGF-D and the density of lymphatic vessels were contradictory [34–36]. In our study, similarly to the results of Mohammad et al. and Gisterek et al., an increase in VEGF-D expression has not correlated with patients shorter survival, whereas a more pronounced expression of VEGF-C was associated with patients poor clinical outcome, confirming earlier reports [35, 38].

No relationship has been documented between the intensity of AT-1R expression and densities of blood and lymphatic vessels (both within the tumour and in the peritumoral area), although a positive correlation has been noted between AT-1R expression and intensities of VEGF-A and VEGF-D expression. Earlier studies of Herr et al. performed on clinical material of breast cancer and on two cellular models of the tumour suggested that Ang II might stimulate production of proangiogenic factors only in cases of triple negative breast cancers [19]. Many controversies have been noted on positive effects of intraepithelial growth factors on processes of angiogenesis and lymphangiogenesis, since the studies were performed on animals and frequently could not be reproduced on the clinical material of ductal breast cancer [39]. Moreover, angiogenic processes are influenced not only by tumour cells but also by tumour-associated fibroblasts and macrophages [40–42].

Our studies have demonstrated no relationship between intensity of AT-1R expression and some clinicopathological parameters, i.e. grade of malignancy, primary tumour size, presence of lymph node metastases, menopausal status or expression of ER, PR and HER2 receptors. In addition, statistical analysis has documented no effects of AT-1R expression intensity on survival of the patients.

Although our results have documented no relationships between AT-1R expression intensity and the listed clinicopathological parameters, in our opinion studies on the role of RAS in development of breast cancer should be continued, taking into account all proteins composing the system. Aside from our results, such a conclusion stems also from recent reports suggesting significant effects of an imbalance between activities of angiotensin-converting enzymes (ACE and ACE2) in the development of breast cancer [43]. This hypothesis was confirmed by studies demonstrating a marked increase in incidence of breast cancer in women carrying D/D allele of the *ACE* gene, which is linked to higher levels of the circulating ACE isoform in blood of these patients [44, 45]. In addition, our results have confirmed effect of AT-1R on expression of VEGF-A. We have detected also a positive correlation between intensities of AT-1R expression and VEGF-D expression in IDC, which may have significance in better understanding the processes of lymphangiogenesis in IDC.
